# Transient Receptor Potential Ankyrin 1 (TRPA1) Is Involved in Upregulating Interleukin-6 Expression in Osteoarthritic Chondrocyte Models

**DOI:** 10.3390/ijms22010087

**Published:** 2020-12-23

**Authors:** Elina Nummenmaa, Mari Hämäläinen, Antti Pemmari, Lauri J. Moilanen, Lauri Tuure, Riina M. Nieminen, Teemu Moilanen, Katriina Vuolteenaho, Eeva Moilanen

**Affiliations:** 1 The Immunopharmacology Research Group, Faculty of Medicine and Health Technology, Tampere University and Tampere University Hospital, FI-33014 Tampere, Finland; elina.nummenmaa@tuni.fi (E.N.); mari.hamalainen@tuni.fi (M.H.); antti.pemmari@tuni.fi (A.P.); lauri.moilanen@tuni.fi (L.J.M.); lauri.tuure@tuni.fi (L.T.); riina.nieminen@tuni.fi (R.M.N.); teemu.moilanen@coxa.fi (T.M.); katriina.vuolteenaho@tuni.fi (K.V.); 2 Coxa Hospital for Joint Replacement, FI-33520 Tampere, Finland

**Keywords:** osteoarthritis, chondrocyte, TRPA1, *IL-6*, inflammation

## Abstract

Transient receptor potential ankyrin 1 (TRPA1) is a membrane-bound ion channel found in neurons, where it mediates nociception and neurogenic inflammation. Recently, we have discovered that TRPA1 is also expressed in human osteoarthritic (OA) chondrocytes and downregulated by the anti-inflammatory drugs aurothiomalate and dexamethasone. We have also shown TRPA1 to mediate inflammation, pain, and cartilage degeneration in experimental osteoarthritis. In this study, we investigated the role of TRPA1 in joint inflammation, focusing on the pro-inflammatory cytokine interleukin-6 (*IL-6*). We utilized cartilage/chondrocytes from wild-type (WT) and TRPA1 knockout (KO) mice, along with primary chondrocytes from OA patients. The results show that TRPA1 regulates the synthesis of the OA-driving inflammatory cytokine *IL-6* in chondrocytes. *IL-6* was highly expressed in WT chondrocytes, and its expression, along with the expression of *IL-6* family cytokines leukemia inhibitory factor (*LIF*) and *IL-11*, were significantly downregulated by TRPA1 deficiency. Furthermore, treatment with the TRPA1 antagonist significantly downregulated the expression of *IL-6* in chondrocytes from WT mice and OA patients. The results suggest that TRPA1 is involved in the upregulation of *IL-6* production in chondrocytes. These findings together with previous results on the expression and functions of TRPA1 in cellular and animal models point to the role of TRPA1 as a potential mediator and novel drug target in osteoarthritis.

## 1. Introduction

Transient receptor potential ankyrin 1 (TRPA1) is a membrane-associated cation channel. TRPA1 is widely expressed in neurons, where it has been shown to mediate pain and neurogenic inflammation and function as a sensor for noxious exogenous compounds [1,2,3,4,5]. TRPA1 has also been shown to be expressed in some non-neuronal cells, such as keratinocytes [6], synoviocytes [7], and very recently also in chondrocytes [8]. The role of TRPA1 in non-neuronal cells is not as clear, but inflammatory effects have been reported [7,8,9]. In addition to being activated by pungent exogenous compounds, TRPA1 is also activated by endogenous factors formed in hypoxic and inflammatory conditions, such as those found in osteoarthritic joints [10,11,12].

Osteoarthritis (OA) is the most common joint disease worldwide, and its prevalence keeps rising as the population ages. OA is a degenerative joint disease characterized by inflammation and hypoxia in the affected joint, which leads to pain, cartilage degeneration and joint deformities [13,14]. The cartilage degeneration in OA is caused by an imbalance between the production of anabolic, catabolic, and inflammatory factors in the affected joint. As the disease progresses, the amount of inflammatory and catabolic mediators increases, while the production of cartilage matrix components decreases [15]. It is not known what initiates this process, but the main inflammatory mediators driving OA pathogenesis are believed to be IL-1β, tumor necrosis factor (TNF)-α, and according to recent studies, particularly *IL-6* [16,17,18]. These cytokines are produced by synovial cells but also by chondrocytes, and they support OA-associated inflammation and cartilage destruction by stimulating the production of matrix degrading enzymes such as matrix metalloproteinases (MMPs) and aggrecanases, as well as pro-inflammatory cytokines and other inflammatory factors including nitric oxide and eicosanoids [15].

We have recently shown that in monosodium iodoacetate (MIA)-induced experimental osteoarthritis, TRPA1 activation has a role in mediating inflammation, cartilage degradation and joint pain [19]. We have also shown that TRPA1 is expressed in primary human osteoarthritic chondrocytes, and that its expression is downregulated by the anti-inflammatory drugs aurothiomalate and dexamethasone [8,20]. In addition to OA, TRPA1 is also implicated in the development of other inflammatory conditions and joint diseases, such as gout and chronic arthritis [21,22,23,24,25,26].

Based on previous findings, TRPA1 mediates joint pain, inflammation, and cartilage destruction in an experimental model of OA [19], is expressed in osteoarthritic chondrocytes [8], and is activated by factors present in inflammatory and hypoxic conditions [10,11,27]. The aim of the present study was to investigate in chondrocytes the role of TRPA1 in joint inflammation, focusing specifically on *IL-6*, which has recently been shown to be essential in driving the pathogenesis of OA.

## 2. Results

### 2.1. TRPA1 Regulates IL-6 Expression in Chondrocytes Based on RNA-Seq Data

We investigated the effect of TRPA1 on *IL-6* by using chondrocytes from TRPA1 deficient (KO) and corresponding wild-type (WT) mice. In next generation RNA sequencing analysis, the pro-inflammatory cytokine *IL-6* was highly expressed in IL-1β-stimulated chondrocytes from WT mice (reads per kilobase per million (RPKM) 202.37), and the expression was significantly lower in chondrocytes from TRPA1 KO mice (RPKM 48.26, Table 1).

We also found that two other members of the *IL-6* cytokine family, namely *IL-11* and leukemia inhibitory factor (*LIF*), were expressed at a significantly higher level in WT chondrocytes compared to TRPA1 KO chondrocytes, although their expression levels in general were lower than those of *IL-6*. The results of the RNA-Seq analysis on *IL-6*, *IL-11* and *LIF* were verified by quantitative RT-PCR analysis (Table 1).

### 2.2. Expression of IL-6 Family Cytokines Is Downregulated by Genetic Deletion and Pharmacological Inhibition of TRPA1

**Figure 1:**
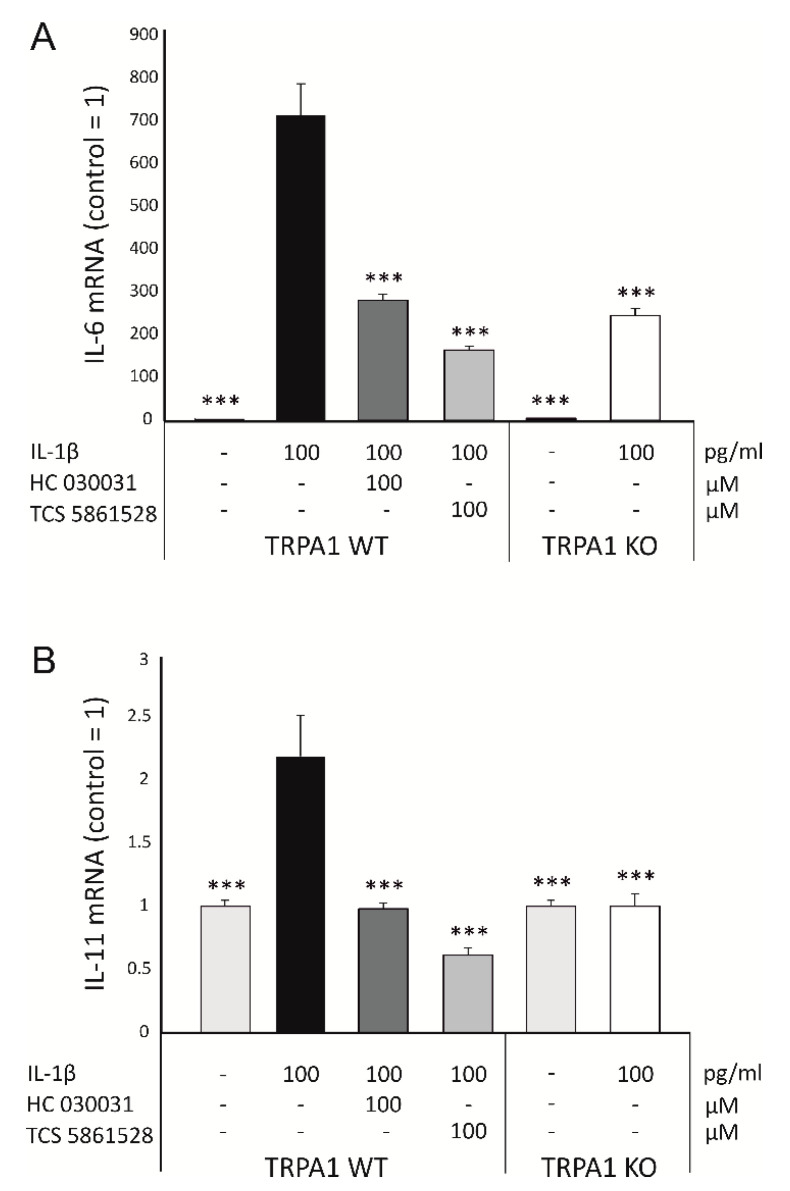
IL-1β-induced *IL-6* (**A**) and *IL-11* (**B**) expression is attenuated by genetic deletion and pharmacological inhibition of TRPA1 in murine chondrocytes. Chondrocytes were obtained from TRPA1 deficient (knockout, KO) mice and corresponding wild-type (WT) mice. The chondrocytes were cultured with IL-1β (100 pg/mL) alone, or together with the selective TRPA1 antagonist HC-030031 (100 μM) or TCS 5861528 (100 μM) for 24 h and thereafter total RNA was extracted. *IL-6* and *IL-11* mRNA levels were measured with qRT-PCR and normalized against glyceraldehyde 3-phosphate dehydrogenase (GAPDH) mRNA levels. The results are expressed as fold change in comparison to the control samples of each genotype. WT *n* = 13 (TCS treatment *n* = 10), KO *n* = 15. Results are expressed as mean + SEM. One-way ANOVA followed by Bonferroni post-test was performed; *** *p* < 0.001. (Adapted from the doctoral dissertation of the first author [28]).

Next, we wanted to confirm the findings in a larger set of chondrocytes from WT and TRPA1 KO mice. As shown in Figure 1A, IL-1β-induced expression of *IL-6* in chondrocytes from TRPA1 deficient mice was significantly lower than that in cells from WT mice. IL-1β treatment also upregulated the expression of *IL-11* in chondrocytes from WT mice, but this effect was not seen in chondrocytes from TRPA1 KO mice (Figure 1B). Additionally, the TRPA1 antagonists TCS 5861528 and HC-030031 significantly attenuated both *IL-6* and *IL-11* expression in chondrocytes from WT mice (Figure 1), indicating that TRPA1 is indeed involved in regulating the expression of these cytokines in murine chondrocytes.

**Figure 2:**
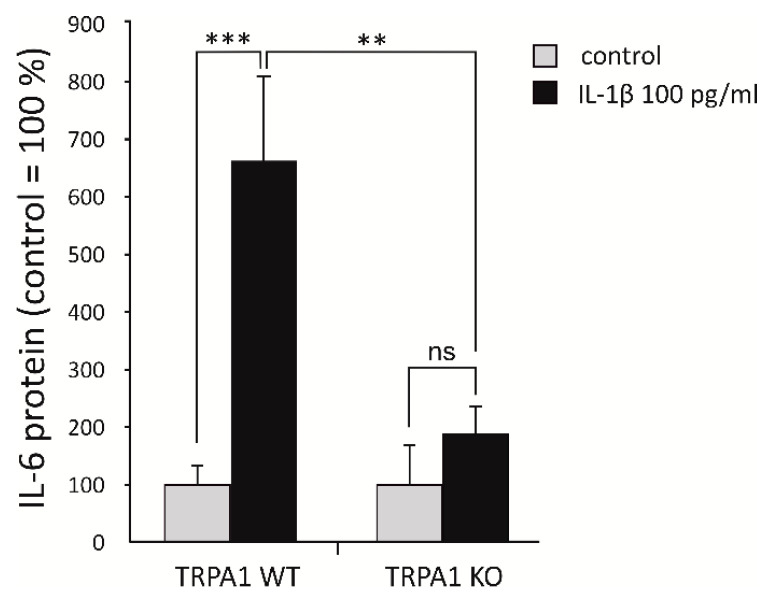
IL-1β-induced production of *IL-6* in cartilage is attenuated by genetic deletion of TRPA1. Cartilage samples (from femoral heads) were obtained from six TRPA1 deficient (KO) mice and six corresponding wild-type (WT) mice. The cartilage pieces were cultured in the presence of IL-1β (100 pg/mL) or without stimulation for 42 h, after which the culture medium was collected, and *IL-6* was measured by immunoassay. The results are expressed as mean + SEM, *n* = 6. One-way ANOVA followed by Bonferroni post-test was performed; ** *p* < 0.01, *** *p* < 0.001, ns: not significant. (Adapted from the doctoral dissertation of the first author [28]).

We also investigated the effect of TRPA1 on *IL-6* production in cartilage explants obtained from WT and TRPA1 KO mice. IL-1β treatment significantly increased *IL-6* production in WT cartilage. This effect was significantly decreased in cartilage from TRPA1 KO mice, where IL-1β treatment did not induce any statistically significant elevation of *IL-6* production (Figure 2).

**Figure 3:**
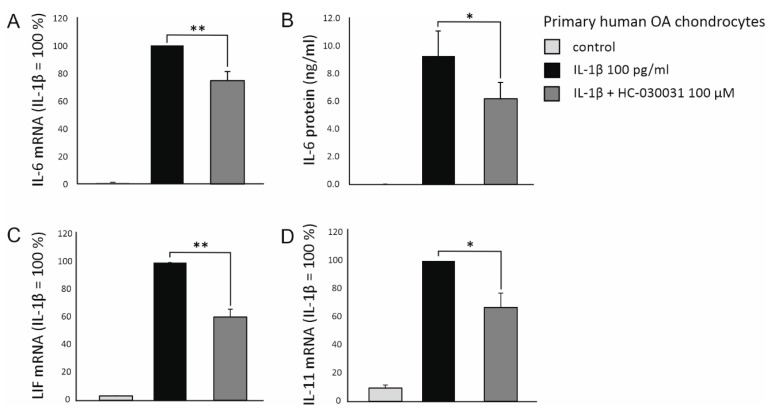
IL-1β-enhanced expression of *IL-6* (**A**,**B**), leukemia inhibitory factor (*LIF*) (**C**) and *IL-11* (**D**) in primary human osteoarthritic (OA) chondrocytes is attenuated by pharmacological inhibition of TRPA1. Primary human OA chondrocytes were stimulated with IL-1β (100 pg/mL) in the presence and absence of the selective TRPA1 antagonist HC-030031 (100 μM) for 24 h, after which the cell culture media were collected, and total RNA was extracted. *IL-6* was measured from the cell culture medium with immunoassay, and *IL-6*, *LIF* and *IL-11* mRNA was measured by qRT-PCR and normalized against *GAPDH* mRNA levels. The results are expressed as mean + SEM, *n* = 7. Samples were obtained from seven patients and the experiments were carried out in duplicate. Paired t-test was used in the statistical analysis; * *p* < 0.05 and ** *p* < 0.01. (Adapted from the doctoral dissertation of the first author [28]).

To verify the results obtained in murine cells, the effect of TRPA1 on the expression of *IL-6*, *IL-11* and *LIF* was also investigated in primary human OA chondrocytes. In accordance with the murine studies, the selective TRPA1 antagonist HC-030031 significantly decreased the IL-1β-enhanced expression of *IL-6*, *IL-11* and *LIF* also in human OA chondrocytes, suggesting that TRPA1 is involved in regulating the expression of these OA-related cytokines also in human OA cartilage (Figure 3).

### 2.3. Possible Mediators Involved in TRPA1-Dependent IL-6 Expression

To preliminarily understand the mechanisms linking TRPA1 and *IL-6*, we examined the Gene Ontology (GO) term “Positive regulation of interleukin-6 production” (GO:0032755) and found it to be significantly altered in chondrocytes from TRPA1 KO mice compared to corresponding WT mice. Therefore, we decided to investigate the genes of this term more closely. Out of the genes included in the GO:0032755 term, expression of receptors *TLR2, CCR5, CD36* and *P2RX7*, adaptor protein *CARD9* and cytokine *IL-33* was significantly downregulated by TRPA1 deficiency both in the RNA-Seq and qRT-PCR analysis (Table 2), and therefore may explain the difference in *IL-6* expression between the chondrocytes of TRPA1 KO and WT mice. (All genes involved in the GO:0032755 term are listed in the Mouse Genome Informatics (MGI) database (http://www.informatics.jax.org/go/term/GO:0032755), and genes involved in the GO:0032755 term with significantly altered expression between chondrocytes from TRPA1 KO and corresponding WT mice are listed in Supplementary Table S1.)

## 3. Discussion

In the present study, we discovered that TRPA1 is involved in the upregulation of *IL-6* family cytokines *IL-6*, *IL-11* and *LIF* in chondrocytes. The finding was confirmed in mouse and human chondrocytes by utilizing selective TRPA1 antagonists, chondrocytes, and cartilage from TRPA1 deficient (KO) and WT mice, along with primary human OA chondrocytes. However, the detailed mechanisms by which TRPA1 mediates *IL-6* expression require further research. The finding is of particular interest because it reveals that the recently recognized inducible expression of TRPA1 in human osteoarthritic chondrocytes is involved in increased *IL-6* production, characteristic for the pathogenesis of arthritis.

In recent years, *IL-6* has emerged as a major player in the pathogenesis of OA. It has long been regarded as an important mediator in rheumatoid arthritis, and recent findings have shown its importance also in osteoarthritis [18,29,30]. *IL-6* is produced by human articular chondrocytes [29,31], and found at elevated concentrations in the synovial fluid and serum of OA patients [32]. The concentrations of *IL-6* in serum and synovial fluid correlate positively with cartilage loss and incidence, or severity, of radiographic knee OA [29,33,34,35]. Notably, the concentration of *IL-6* in the synovial fluid of OA patients significantly exceeds that of IL-1β, supporting the role of *IL-6* in the pathogenesis of OA [36]. The *IL-6* family cytokines *LIF* and *IL-11* are also implicated in arthritis [15,37]. Elevated levels of *LIF* are found in the synovial fluid and cartilage of OA patients, and it is associated with pro-inflammatory and catabolic responses [17,37,38,39]. *IL-11* is found in the serum, synovial fluid, and cartilage of OA patients, and it has been shown to have both pro- and anti-inflammatory properties [37,40,41].

The effects of *IL-6* in cartilage include upregulation of matrix degrading enzymes MMP-1 and MMP-13 and downregulation of the major extracellular matrix component type II collagen [16]. *IL-6* is also considered to be the key cytokine causing changes in subchondral bone, primarily via promotion of osteoclast formation [42]. Considering the effects of *IL-6* in OA joints, it appears to be a promising drug target. Accordingly, the *IL-6* receptor antagonist tocilizumab is currently in phase III clinical trials for pain and function in patients with refractory hand OA (NCT02477059) [43].

Another approach to drug treatment could be to target the expression and production of *IL-6*. In this study, *IL-6* expression was shown to be regulated by TRPA1. In support of the current findings, we have recently shown in experimental monosodium urate (MSU) crystal-induced gouty inflammation that the production of *IL-6* was attenuated in TRPA1 KO mice and by pharmacological inhibition of TRPA1 [21]. TRPA1 has also been reported to mediate *IL-6* synthesis in the peritoneal tissue in the rat in response to peritoneal dialysis fluid administration [44]. Further, in an oxazolone-induced atopic dermatitis model, the expression of *IL-6* was significantly decreased in skin samples from TRPA1 KO mice compared to WT mice [45]. Based on the results of this paper, and previous findings on the effects of TRPA1 on *IL-6* production, inhibition of the expression and/or activation of TRPA1 in osteoarthritic cartilage could downregulate the production of *IL-6* and other members of the *IL-6* cytokine family. In addition, TRPA1 inhibitors are likely to have analgesic effects on arthritis pain. Of note, TRPA1 antagonist GRC 17536 has been studied in a phase II clinical trial for painful diabetic neuropathy (NCT01726413) [46].

The effects of TRPA1 are predominantly attributed to the influx of Ca^2+^ through the activated channel and the subsequent increase in intracellular Ca^2+^. Among other functions, the elevation of intracellular Ca^2+^ concentration mediates inflammatory gene expression both directly and indirectly [47]. In this study, we aimed to preliminarily examine which mediators could be involved in TRPA1-dependent *IL-6* production by utilizing RNA-Seq data. We focused specifically on genes that according to the GO term “Positive regulation of interleukin-6 production” (GO:0032755) relate to *IL-6* production. Of these, genes that could explain the difference in *IL-6* expression between the chondrocytes of TRPA1 KO and corresponding WT mice include receptors *TLR2*, *CCR5*, *CD36* and *P2RX7*, adaptor protein *CARD9* and cytokine *IL-33*. These genes were all expressed at lower levels in chondrocytes from TRPA1 KO mice compared to WT mice (Table 2), and they have previously been reported to increase the production of *IL-6* in inflammatory cell types and/or cells present in the joint [48,49,50,51,52,53]. *TLR2* has been found to increase *IL-6* production in septic arthritic chondrocytes [48], and *CCR5* activation has been reported to increase *IL-6* expression in osteoarthritic synoviocytes [49]. *CD36* and *CARD9* are involved in *IL-6* expression in dendritic cells [50,52], and *IL-33* has been shown to induce *IL-6* production in mast cells [53]. In addition to cells of the immune system, *CD36* and *IL-33* are also found in human OA cartilage and rheumatoid arthritis (RA) synovium, respectively [54,55].

Particularly interesting with regard to TRPA1 function is the receptor *P2RX7*, which is an adenosine triphosphate (ATP)-activated ion channel linked to inflammation and noxious cold sensation [51]. ATP is released from stressed and damaged cells during inflammation [56], and its release can be mediated by TRPA1 [57]. It has also been suggested that the channel function of P2X7 receptors may affect TRPA1 channels [58]. Further, activation of *P2RX7* induced *IL-6* production in human fibroblasts [59]. However, further studies are needed to reveal the mediator role of the genes listed in Table 2, as well as to uncover the more detailed mechanisms by which TRPA1 mediates the production of *IL-6*, *LIF* and *IL-11* in chondrocytes.

In conclusion, we found in the present study that the expression of the OA-driving factor *IL-6*, along with *IL-6* family cytokines *LIF* and *IL-11*, is upregulated by TRPA1 in chondrocytes. The role of TRPA1 in the production of *IL-6* in cartilage/chondrocytes may be essential in the pathogenesis and inflammatory processes in OA. These results, together with the previous findings on the expression and functions of TRPA1 in OA inflammation and pain, support TRPA1 as a potential factor and novel drug target in OA.

## 4. Materials and Methods

### 4.1. Animals

Whole body TRPA1 knockout (KO) B6;129P-Trpa1 ^tm1Kykw^/J and corresponding wild-type (WT) mice (Charles River Laboratories, Sulzfeld, Germany) aged 6–14 days were used in mouse chondrocyte culture experiments and aged 19–22 days for cartilage culture experiments. The TRPA1 KO mice have been developed by disrupting the S5 and S6 transmembrane domains and the interjacent TRPA1 pore loop [60]. The mice were kept in standard conditions (22 ± 1 °C temperature, 12–12 h dark–light cycle, water and food provided ad libitum). The animals were housed and handled according to the legislation for the protection of animals used for scientific purposes (Directive 2010/63/EU, 22 September 2010).

### 4.2. Mouse Cartilage and Chondrocyte Culture

After euthanization of the mice, full-thickness femoral head articular cartilage was removed and used for cartilage and chondrocyte culture experiments. For the cartilage culture experiments, femoral heads were extracted from six WT and six TRPA1 KO mice. The cartilage samples were washed with phosphate buffered saline (PBS) and then incubated at 37 °C in 5% CO_2_ in Dulbecco’s Modified Eagle Medium (DMEM) supplemented amphotericin B (250 ng/mL), streptomycin (100 μg/mL) and penicillin (100 U/mL, all from Gibco/Life Technologies, Carlsbad, CA, USA) containing 10 % fetal bovine serum (FBS). The cartilage pieces were treated with IL-1β (100 pg/mL, R&D Systems Europe Ltd., Abingdon, UK) for 42 h, after which the culture media were collected, and *IL-6* levels were determined by immunoassay.

Mouse chondrocytes were isolated as previously described [61]. For the chondrocyte culture experiments, chondrocytes from a total of 40 WT mice and 47 TRPA1 KO mice were isolated from femoral head cartilage samples by enzymatic digestion for 16 h at 37 °C in 5% carbon dioxide using the collagenase D enzyme (3 mg/mL, Sigma-Aldrich, St. Louis, MO, USA), following the protocol published by Jonason et al. [62]. Extracted chondrocytes were seeded on 24-well plates (2.0 × 10^5^ cells/mL) in cell culture medium (DMEM high glucose (Sigma-Aldrich) supplemented with amphotericin B (250 ng/mL), streptomycin (100 μg/mL) and penicillin (100 U/mL, all from Gibco/Life Technologies) containing 10% fetal bovine serum (FBS) (Lonza, Verviers, Belgium)). The chondrocytes were cultured for seven days prior to conducting the experiments, and in the experiments, the chondrocytes were treated for 24 h with IL-1β (R&D Systems Europe Ltd.), the TRPA1 antagonists TCS 5861528 (Tocris, Bio-Techne Ltd., Abingdon, UK), HC-030031 (Sigma-Aldrich) or with their combination as indicated.

### 4.3. Human Chondrocyte Culture

Primary human OA chondrocytes were isolated from leftover pieces of osteoarthritic cartilage from total knee replacement surgery of seven OA patients. The patients in this study fulfilled the European League Against Rheumatism (EULAR) classification criteria for knee OA [63]. The study was performed in accordance with the Declaration of Helsinki and approved by the ethics committee of Tampere University Hospital, Tampere, Finland. All patients gave their written informed consent. Isolation and culture of human chondrocytes were performed as previously described [64]. Articular cartilage was removed aseptically from subchondral bone, cut into pieces, and washed with PBS. After which, the chondrocytes were enzymatically isolated by incubating the cartilage pieces in the presence of Liberase^TM^ enzyme (0.25 mg/mL, Roche, Mannheim, Germany) for 16 h at 37 °C in a shaker. The extracted chondrocytes were washed and seeded on 24-well plates (2.0 × 10^5^ cells/mL) in DMEM high glucose (Sigma-Aldrich) culture medium supplemented with amphotericin B (250 ng/mL), streptomycin (100 μg/mL) and penicillin (100 U/mL, all from Gibco/Life Technologies) containing 10 % fetal bovine serum (FBS) (Lonza, Verviers, Belgium). The chondrocytes were cultured for 24 h prior to conducting experiments, and in the experiments, the chondrocytes were treated for 24 h with IL-1β (R&D Systems Europe Ltd.), the TRPA1 antagonists HC-030031 (Sigma-Aldrich) or with their combination as indicated.

### 4.4. RNA Extraction and Sample Preparation

Culture medium was collected, and total RNA of the chondrocytes was extracted at the indicated time points. Total RNA from primary human OA chondrocytes was extracted with GenElute Mammalian Total RNA Miniprep Kit (Sigma-Aldrich), and the concentration of RNA was determined with a spectrophotometer. Total RNA from murine chondrocytes was extracted with RNeasy Mini Kit (QIAGEN Inc., Hilden, Germany) and treated with RNase-Free DNAse Set (QIAGEN Inc.). The concentration of RNA and integrity of the samples was measured with 2100 Bioanalyzer (Agilent Technologies Inc., Santa Clara, CA, USA).

### 4.5. Next-Generation RNA Sequencing (RNA-Seq) and Data Analysis

Samples for RNA-Seq were prepared by pooling chondrocytes from 11 TRPA1 deficient (KO) mice and 12 corresponding WT mice. Four samples obtained from the pool of WT mice and five samples from the pool of TRPA1 KO mice were sequenced. In addition, results from RNA-Seq were verified with quantitative RT-PCR in a larger set of samples. The samples were sequenced at the Finnish Institute of Molecular Medicine (FIMM), Helsinki, Finland. The Illumina HiSeq 2500 System was used with a sequencing depth of 20 M paired-end reads, 100 bp in length. Read quality was evaluated using FastQC [65]. The reads were trimmed with Trimmomatic [66] and aligned to reference mouse genome with Spliced Transcripts Alignment to a Reference (STAR) [67]. The featureCounts program was used to prepare count matrices [68]. Gene expression levels were determined as reads per kilobase per million (RPKM) [69]. Differential gene expression was assessed with DESeq2 [70], and the Database for Annotation, Visualization and Integrated Discovery (DAVID) was used to perform functional analysis [71].

### 4.6. Quantitative RT-PCR

Quantitative RT-PCR (qRT-PCR) was performed as previously described [72]. Total RNA was extracted as described above, and reverse transcribed to cDNA using TaqMan Reverse Transcription reagents (Applied Biosystems, Foster City, CA, USA) or Maxima First Strand cDNA synthesis kit (Fermentas UAB, Vilnius, Lithuania). qRT-PCR was performed with TaqMan Universal PCR Master Mix and the ABI 7500 Real-Time PCR system (Applied Biosystems). The probe and primer sequences (Table 3) and concentrations for mouse and human *IL-6* and glyceraldehyde 3-phosphate dehydrogenase (*GAPDH*) were designed with Primer Express^®^ Software (Applied Biosystems) and optimized following the manufacturer’s guidelines. The primers were purchased from Metabion (Martinsried, Germany). TaqMan Gene Expression assays for human leukemia inhibitory factor (*LIF*) (Hs01055668_m1), mouse *LIF* (Mm00434762_g1), human *IL-11* (Hs01055414_m1) and mouse *IL-11* (Mm00434162_m1) were obtained from Life Technologies (Life Technologies Europe BV, Bleiswijk, the Netherlands). The relative mRNA levels of the genes listed in Table 3 were determined using a standard curve method. The ΔΔCt method was used for the TaqMan Gene Expression assays, and all mRNA expression levels were normalized against *GAPDH*.

### 4.7. Immunoassay

Concentrations of *IL-6* in medium samples were measured by enzyme-linked immunosorbent assay (ELISA) with commercial reagents (human *IL-6*: eBioscience Inc. San Diego, CA, USA; mouse *IL-6*: R&D Systems Europe Ltd.).

### 4.8. Statistics

GraphPad InStat 3.00 software (GraphPad Software, San Diego, CA, USA) was used for data analysis. Data are presented as mean + standard error of the mean (SEM). Paired t-test or one-way analysis of variance (ANOVA), followed by Bonferroni post-test, were used in the statistical analysis where appropriate. False discovery rate (FDR)-corrected *p*-values were used in the RNA-Seq-analysis. Differences were considered significant at * *p* < 0.05, ** *p* < 0.01, and *** *p* < 0.001. For a detailed description of the RNA-Seq data analysis, please refer to Section 4.5.

## Figures and Tables

**Table 1:** *IL-6* cytokine family members showing attenuated expression in chondrocytes obtained from transient receptor potential ankyrin 1 (TRPA1) deficient (KO) mice compared to chondrocytes from corresponding wild-type (WT) mice.

Gene		RNA-Seq				qRT-PCR	
		RPKM (WT)	RPKM (TRPA1 KO)	FC	Adj. *p*-Value	FC	Adj. *p*-Value
*IL-6*	Interleukin-6	202.37	48.26	−3.86	<0.0001	−8.93	<0.001
*LIF*	Leukemia inhibitory factor	12.03	5.19	−2.16	<0.0001	−3.95	<0.001
*IL-11*	Interleukin-11	1.11	0.21	−4.14	<0.0001	−4.56	<0.001

RNA-Seq analysis was performed on IL-1β-treated chondrocytes obtained from TRPA1 deficient (KO) and corresponding wild-type (WT) mice. Gene expression levels are given as RPKM. Differences between the genotypes are given as fold change (FC) values, with negative values indicating downregulated genes in chondrocytes from TRPA1 KO mice. The *p*-values are adjusted by false discovery rate (FDR). The RNA-Seq results were verified by qRT-PCR, where *p*-values are given as Bonferroni-adjusted. (Adapted from the doctoral dissertation of the first author [28]); FDR—false discovery rate, KO—knockout, RPKM—reads per kilobase per million, WT—wild-type.

**Table 2:** Genes putatively explaining the differential expression of *IL-6* production according to Gene Ontology (GO) analysis.

Gene		RNA-Seq				qRT-PCR	
		RPKM (WT)	RPKM (TRPA1 KO)	FC	Adj. *p*-Value	FC	Adj. *p*-Value
*TLR2*	toll-like receptor 2	26.67	12.48	−2.01	<0.0001	−3.92	<0.001
*CD36*	*CD36* antigen	22.39	8.59	−2.41	<0.0001	−3.81	<0.001
*CCR5*	chemokine (C-C motif) receptor 5	13.15	1.70	−6.87	<0.0001	−15.68	<0.001
*P2RX7*	purinergic receptor P2X, ligand-gated ion channel, 7	3.73	1.35	−2.57	<0.0001	−2.9	<0.001
*CARD9*	caspase recruitment domain family, member 9	2.10	0.39	−4.41	<0.0001	−9.04	<0.001
*IL-33*	interleukin-33	0.34	0.06	−2.81	0.0022	−11.6	<0.001

RNA-Seq analysis was performed on IL-1β-treated chondrocytes obtained from TRPA1 deficient (KO) and corresponding wild-type (WT) mice. Gene expression levels are given as RPKM. Differences between the genotypes are given as fold change (FC) values, with negative values indicating downregulated genes in chondrocytes from TRPA1 KO mice. The *p*-values are adjusted by false discovery rate (FDR). The RNA-Seq results were verified by qRT-PCR, where *p*-values are given as Bonferroni-adjusted; FDR—false discovery rate, GO—Gene Ontology, KO—knockout, RPKM—reads per kilobase per million, WT—wild-type.

**Table 3:** Primers and probes used in qRT-PCR.

Primer/Probe		Sequence
*hGAPDH*	forward	5′-AAGGTCGGAGTCAACGGATTT-3′
	reverse	5′-GCAACAATATCCACTTTACCAGAGTTAA-3′
	probe	5′-CGCCTGGTCACCAGGGCTGC-3′
*hIL-6*	forward	5′-TACCCCCAGGAGAAGATTCCA-3′
	reverse	5′-CCGTCGAGGATGTACCGAATT-3′
	probe	5′-CGCCCCACACAGACAGCCACTC-3′
*mGAPDH*	forward	5′-GCATGGCCTTCCGTGTTC-3′
	reverse	5′-GATGTCATCATACTTGGCAGGTTT-3′
	probe	5′-TCGTGGATCTGACGTGCCGCC-3′
*mIL-6*	forward	5′-TCGGAGGCTTAATTACACATGTTC-3′
	reverse	5′-CAAGTGCATCATCGTTGTTCATAC-3′
	probe	5′-CAGAATTGCCATTGCACAACTCTTTTCTCA-3′

GAPDH—glyceraldehyde 3-phosphate dehydrogenase, IL—interleukin, qRT-PCR—quantitative reverse transcription polymerase chain reaction.

## Data Availability

The data presented in this study are available on request from the corresponding author.

## Supplementary Materials

The following are available online at https://www.mdpi.com/1422-0067/22/1/87/s1, Table S1: Genes involved in the GO term “Positive regulation of interleukin-6 production” (GO:0032755) with significantly altered expression in chondrocytes from TRPA1 KO mice compared to cells from corresponding WT mice.

## Abbreviations

AITC	allyl isothiocyanate
ANOVA	analysis of variance
ATP	adenosine triphosphate
*CARD9*	caspase recruitment domain family, member 9
*CCR5*	chemokine (C-C motif) receptor 5
*CD36*	*CD36* antigen
ELISA	enzyme-linked immunosorbent assay
FC	fold change
FDR	false discovery rate
GO	Gene Ontology
IL	interleukin
Lif	leukemia inhibitory factor
MIA	monosodium iodoacetate
MMP	matrix metalloproteinase
MSU	monosodium urate
OA	osteoarthritis
*P2RX7*	purinergic receptor P2X7
qRT-PCR	quantitative reverse transcription polymerase chain reaction
RNA-Seq	RNA sequencing
RPKM	reads per kilobase per million
*TLR2*	Toll-like receptor 2
TNF	tumor necrosis factor
TRPA1	transient receptor potential ankyrin 1
WT	wild-type
