# Supplementary material for: Transient Receptor Potential Ankyrin 1 (TRPA1) Is Involved in Upregulating Interleukin-6 Expression in Osteoarthritic Chondrocyte Models
Source: Int J Mol Sci. 2020 Dec 23;22(1):87. doi: 10.3390/ijms22010087 (PMC7794684; doi:10.3390/ijms22010087)
Supplement: Supplementary file 1 [file ijms-22-00087-s001.pdf]

Supplementary Table S1.

Genes involved in the GO term 'Positive regulation of interleukin-6 production' (GO:0032755) with significantly altered expression in chondrocytes from TRPA1 KO mice compared to cells from corresponding WT mice

GO term	Number of altered genes	Altered genes	FDR-adjusted p-value
Positive regulation of interleukin-6 production (GO:0032755)	14	*IL33, SPON2, TICAM2, CARD9, IL1F9, P2RX7, PYCARD, IL1A, IL6, IL1F6, IL1B, CD36, CCR5, TLR2*	0.0135

Next-generation RNA sequencing was performed on IL-1β-treated chondrocytes from TRPA1 KO and corresponding WT mice. Gene Ontology (GO)-analysis focusing on the GO term 'Positive regulation of interleukin-6 production' (GO:0032755) was performed using the DAVID database. P-value is given as false discovery rate (FDR)-adjusted.

All genes involved in GO:0032755 term according to the Gene Ontology Browser of the Mouse Genome Informatics (MGI) database can be found at: http://www.informatics.jax.org/go/term/GO:0032755
(accessed 13.11.2020)
